# Ramipril attenuates left ventricular remodeling by regulating the expression of activin A-follistatin in a rat model of heart failure

**DOI:** 10.1038/srep33677

**Published:** 2016-09-19

**Authors:** Qun Wei, Haiyan Liu, Miao Liu, Chunyan Yang, Jie Yang, Zhonghui Liu, Ping Yang

**Affiliations:** 1 Department of cardiology, China-Japan Union Hospital, Jilin University, Changchun, China; 2 Department of Cardiology, Tianjin Fourth Central Hospital, Tianjin, China; 3 Department of Anatomy, College of Basic Medical Sciences, Jilin University, Changchun, China; 4 Department of Immunology, College of Basic Medical Sciences, Jilin University, Changchun, China

## Abstract

Prior studies have shown that overexpression of ACT A can lead to ventricular remodeling in rat models of heart failure. Furthermore, recently work studying demonstrated that stimulation of activin An expression in rat aortic smooth muscle (RASM) cells by angiotensin II (Ang II). Ramipril is a recently developed angiotensin converting enzyme (ACE) inhibitor. To investigate the effects of Ramipril on expression of ACT A-FS, we established the rat model of heart failure after myocardial infarction (MI), and divided into either a sham operation (SO), MI, or MI-Ramipril group. We found that Ramipril significantly attenuates collagen-I and III deposition (col-I and III). Notably, we determined that expression of ACT A and II activin receptor (ActRII) were significantly down-regulated in the non-infarcted area of the left ventricle in the Ramipril group, whereas the mRNA and protein levels of FS were markedly up-regulated. Our data suggested that Ramipril benefited left ventricular remodeling by reducing fibrosis and collagen accumulation in the left ventricle of rats after myocardial infarction. This observation was also associated with down-regulation of ACT A expression. This study elucidated a new protective mechanism of Ramipril and suggests a novel strategy for treatment of post-infarct remodeling and subsequent heart failure.

Activin A (ACT A) is a member of the transforming growth factor-β superfamily, as a kind of pre-inflammatory factor, it has attracted more attention in recent years. In particular, activin A is associated with fibrosis, with several experiments confirming that activin A is an important factor of inducing fibrosis of liver of animals^1^,^2^,^3^. Follistatin (FS) is an activin-binding protein that is measurable in extracellular matrix and serum^4^,^5^. When Activins bind to FS, the complex is internalized by endocytosis and are subsequently degraded by proteolysis^6^. Prior studies have shown that the expression of ACT A and its receptors in ischemic and non-ischemic regions markedly increased after myocardial infarction (MI). Further, ACT A promotes gene expression of atrial natriuretic peptide (ANP), brain natriuretic peptide (BNP) and matrix metalloproteinase-9 (MMP-9), which were known to be factors playing an important role in myocardial remodeling and interstitial fibrosis^7^. The co-expression of ACT A and FS is vital in tissue remodeling and repair, and an imbalance in ACT A-FS expression may induce adverse pathological changes. Elevated activin βAmRNA and activin A protein levels and, to a lesser extent, activin βB mRNA levels were found not only in healing mouse skin wounds but also in human wounds^8^,^9^. In acute mouse and human wounds, keratinocytes of the hyperproliferative wound epidermis as well as fibroblasts and inflammatory cells of the granulation tissue were identified as sources of activin^2^. Furthermore, transgenic mice overexpressing either activin or follistatin in the basal layer of the epidermis were generated^10^,^11^. Overexpression of activin enhanced granulation tissue formation and reepithelialization, but also caused excessive scarring. In contrast, overexpression of follistatin had the opposite effect and caused a delay in repair. These studies implicate an important function of endogenous activin in wound repair and scar formation. FS, an activin antagonist, may prevent ACT A-induced fibrosis and play an important role in the treatment of fibrosis.

In this study we hypothesized that an imbalance in ACT A-FS expression is involved in myocardial fiborsis and subsequent heart failure (HF) after MI. To address this hypothesis, we analyzed the relationship between the imbalance of ACT A-FS expression and left ventricular remodeling in a rat model of heart failure after MI. Since an enormous amount of research and clinical experience have confirmed the ability of ACE inhibitors to reduce morbidity and mortality in patients with MI^12^,^13^ and heart failure, we also investigated the effects of Ramipril on expression of ACT A-FS and regulation of collagen synthesis in the noninfarcted area of the left ventricle. In the current study we report a novel mechanism that can explain the cardiac antifibrotic effect of ACE inhibitors and subsequent protective effect on left ventricular remodeling.

## Results

To investigate the effects of Ramipril on expression of ACT A -FS, we established a rat model of heart failure after MI and divided rats into either a SO, SO-Ramipril, MI, or MI-Ramipril group. All treatment regimens were initiated 7 weeks after surgery and continued for 5 weeks.

### Morphological analysis of HF

**Figure 1:**
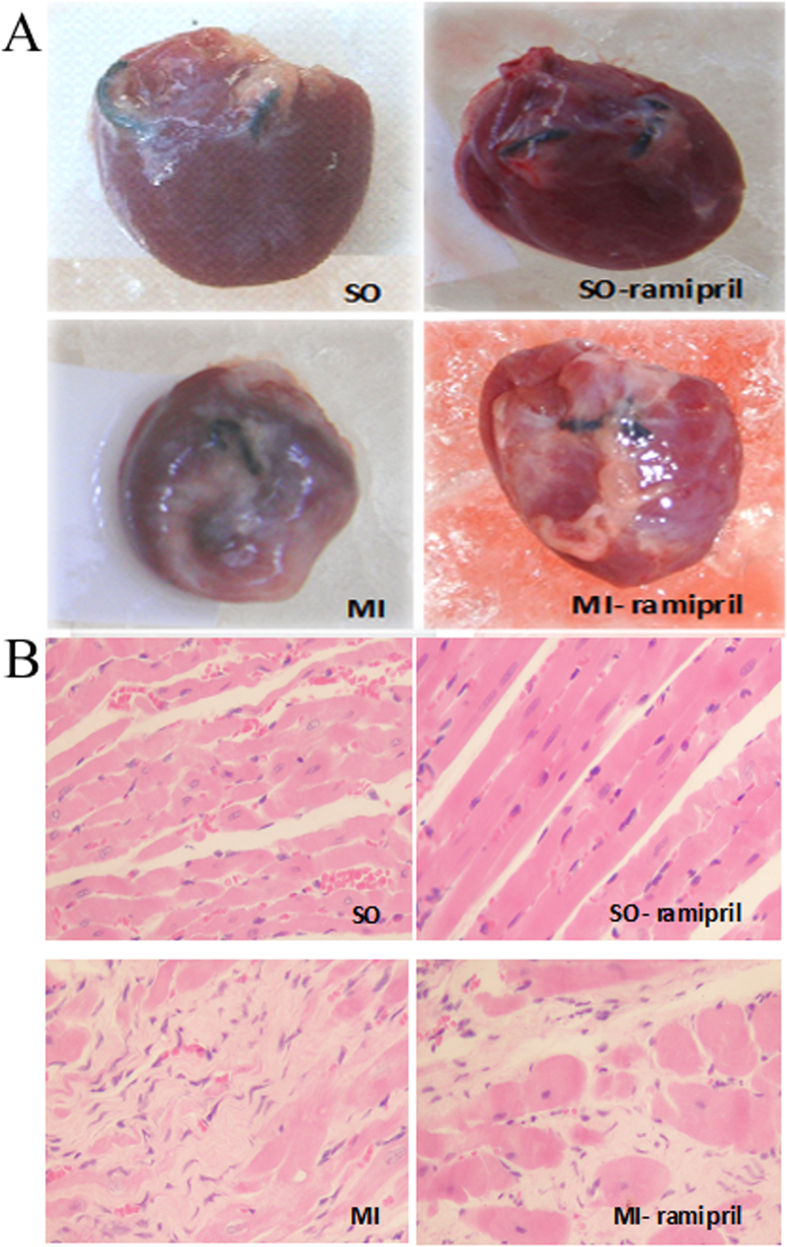
Morphology of rat hearts. (**A**) Gross morphology of hearts of sham-operated rats (SO), ramipril-treated SO rats (SO-ramipril), rats with myocardial infarction (MI) and ramipril-treated MI rats. (**B**) Hematoxylin and eosin staining of the myocardial tissue in the non-infarcted area of the left ventricle.

The heart volume, ventricular chamber size, and ventricular wall thickness were within the normal ranges in SO and SO-ramipril groups, with the heart characterized as an ellipsoid shape, mooth surface, and good elasticity. In MI and MI-ramipril groups, the heart volume markedly increased and the ventricular wall was significantly thinner in the infarcted area as compared with non-infarcted area. In the infarcted area, there was the presence of fibrosis and scar formation, the surface was not smooth, and there was an absence of elasticity. The heart was pale, and the boundary between infarcted and non-infarcted areas was pale-red. Ventricular aneurysm presented in the most infarcted area, which was spherical *in vivo* and collapsed into the ventricular chamber after excision of the heart. The ventricular chamber size significantly increased after MI, and the heart showed eccentric hypertrophy. Compared with the MI group, the heart volume, the infarct size, and the incidence of ventricular aneurysm significantly decreased in the MI-ramipril group (Fig. 1A). H&E staining showed that the cardiomyocytes were intact and the arrangement of myocardial fibers was regular in the non-infarcted area of the left ventricle of SO and SO-ramipril groups (Fig. 1B). In the MI group, the arrangement of myocardial fibers was irregular in the non-infarcted area of the left ventricle, and there was various degrees of cardiomyocyte degeneration and hypertrophy and interstitial fibrosis. Compared with the MI group, the above changes were less severe in the MI-ramipril group.

### Cardiac function

Table 1 shows the rat morphological and hemodynamic characteristics. HW/BW and LVM/BW were significantly higher in the MI group than in the SO group (both *P* < 0.001) 11 weeks after surgery, and they were also significantly higher in MI-ramipril group as compared to the MI group (*P* < 0.01). Compared with the SO group, SBP, DBP, LVSP, and ±dp/dt were significantly decreased and LVEDP was significantly increased in the MI group. In the MI-ramipril group, LVSP, +dp/dt and −dp/dt were significantly higher and LVEDP was significantly lower, as compared with the MI group; however, no significant differences were observed in HR, SBP and DBP.

### *Expressions of ACT A,* ActRII, FS and col I*, III mRNA in the noninfarcted area of the left ventricle*

**Figure 2:**
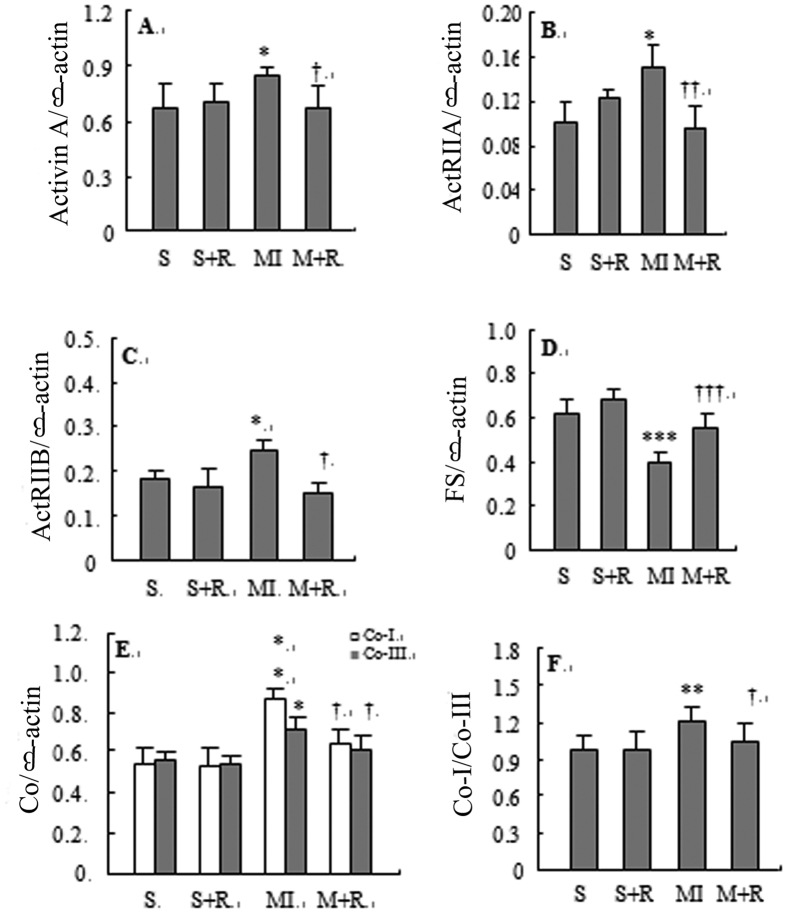
Expression of activin A (panel A), ActRIIA (panel B), ActRIIB (panel C), follistatin (panel D), collagen I and collagen III mRNA (panel E), and the ratio of col I to col III (panel F) in the non-infarcted area of the left ventricle detected by semi-quantitative reverse transcription polymerase chain reaction. S represented SO group, S + R represented SO-ramipril, MI represented MI-model, M + R represented MI-ramipril. The depicted data are the means ± SD. *P < 0.05, **P < 0.01, ***P < 0.001 vs S; ^†^*P* < 0.05, ^††^*P* < 0.01, ^†††^*P* < 0.001vs MI.

The expression levels of ACT A, ActRIIA, ActRIIB, col I, and col III mRNA in the non-infarcted area of the left ventricle and the ratio of col I to col III were higher in MI group than in SO group; however, the expression level of FS mRNA in the MI group was significantly lower compared with the SO group (Fig. 2). Compared with the MI group, the expression of ACT A, ActRIIA, ActRIIB, col I, and col III mRNA and the ratio of col I to col III were lower; however, the expression level of FS mRNA was higher in the MI-ramipril group.

### Level of ACT A, AngII, and BNP in sera of HF rats

The serum levels of ACT A, Ang II, and BNP in the MI group were significantly higher that the SO group, and compared with the MI group, the levels were significantly attenuated in the MI-ramipril group (Table 2). Notably, in the MI-ramipril group, the expression of these 3 proteins was down-regulated (Table 2).

### Levels of ACT A, FS, and Ang II in the noninfarcted area of the left ventriucle

The levels of ACT A and Ang II in the MI group were significantly higher than those in the SO group 11 weeks after surgery; however, the levels of FS were significantly lower in the MI group compared with the SO group (Table 2). Compared with the MI group, the expression of ACT A and Ang II were down-regulated and the expression of FS was up-regulated in the MI-ramipril group.

### Expression of ACT A and FS in HF myocardium

**Figure 3:**
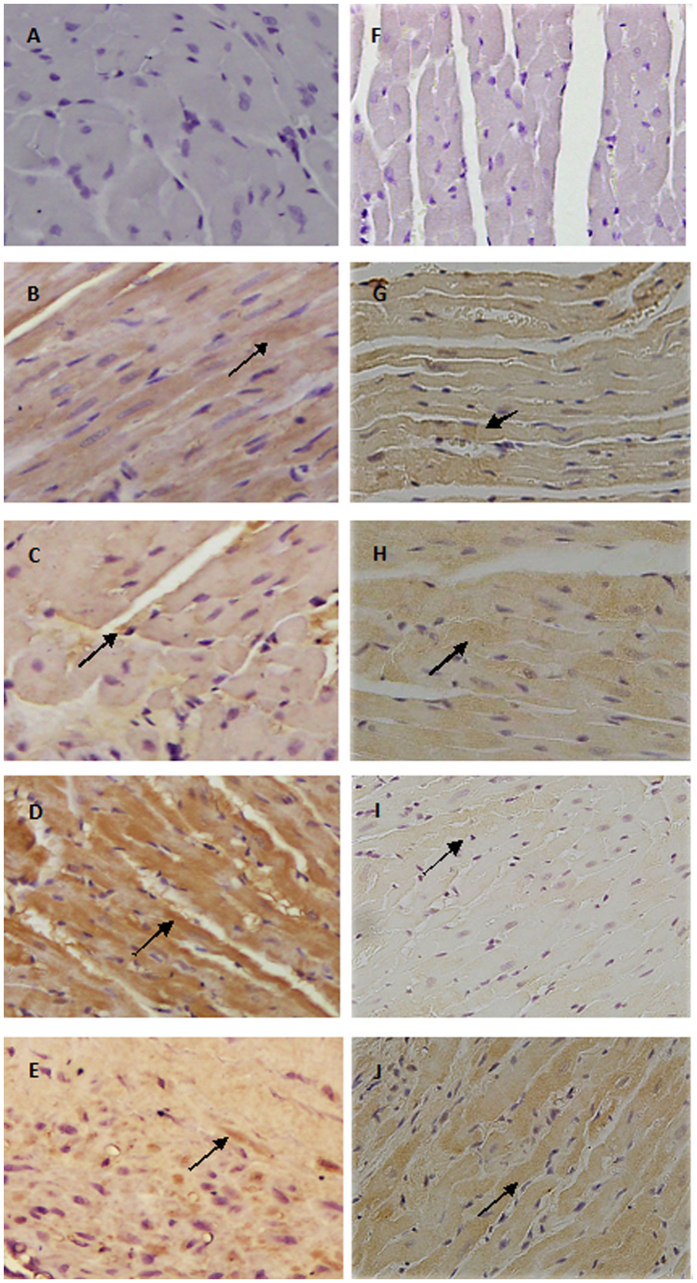
Immunohistochemical staining for activin A and follistatin using anti-activin A antibody (**B–E**) and anti-follistatin antibody (**G–J**) in the non-infarcted area of the left ventricle. (**A,F**) represented procedural background control using normal rabbit IgG. (**B,H**) represented SO group, (**C,H**) represented SO-ramipril group, (**D,I**) represented MI-model group, (**E,J**) represented MI-ramipril group (200× magnification).

ACT A and FS expression in the non-infarcted area of the left ventricle were detectable in SO and SO-ramipril groups (Fig. 3). In the MI group, the expression of ACT A was up-regulated compared with the SO group, and the expression of FS was down-regulated, while the converse was true in the MI-ramipril group.

### Myocardial fibrosis after MI

**Figure 4:**
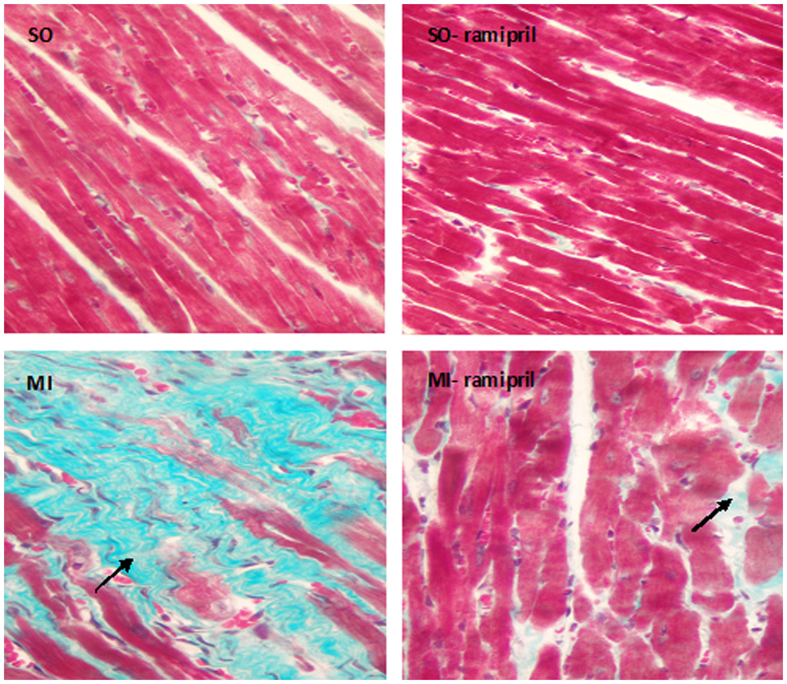
Myocaridal fibrosis after myocardial infarction detected by Masson’s trichrome staining in sham-operated rats (SO), ramipril-treated SO rats (SO-ramipril), rats with myocardial infarction (MI) and ramipril-treated MI rats in the noninfarcted area of the left ventricle (200× magnification).

Masson’s trichrome stain revealed the formation of myocardial fibrosis in the non-infarcted area of the left ventricle after MI. In SO and SO-ramipril groups, cardiomycyte arrangement was regular, and no obvious green collagen deposition was identified (Fig. 4). In the MI group, collagen fiber deposition and cardiomyocyte hypertrophy were detected, the number of cardiomyocytes decreased, and the arrangement of cardiomyocytes was irregular. Collagen deposition in the MI-ramipril group was lower as compared with the MI group.

## Discussion

Prior studies have confirmed that the imbalance of the ACT A-FS system in a rat model of HF causes an up-regulation of ACT A and down-regulation of FS^7^,^14^. To investigate the effects of Ramipril on expression of ACT A-FS, we established a rat model of heart failure after MI and divided rats into either a SO, SO-Ramipril, MI, or MI-Ramipril group. All treatment regimens were initiated 7 weeks after surgery and continued for 5 weeks. Consistent with these results, the current study showed that expression of ACT A, ActRIIA, and ActRIIB mRNA in the non-infarcted area of the left ventricle were significantly higher in the MI group compared with the SO group. ELISA confirmed that ACT A protein expression in the non-infarcted area of the left ventricle were significantly higher in the MI group compared with the SO group. We also found the expression levels of col I and col III mRNA and the ratio of col I to col III significantly increased after MI. Unexpectedly, the expression of FS in the non-infarcted area of the left ventricle was significantly decreased compared with the SO group. In the non-infarcted area of the left ventricle, the up-regulated expression of ACT A and down-regulated expression of FS were confirmed by immunohistochemistry, and collagen deposition was identified by Masson’s trichrome stain.

These results suggested an imbalance in ACT A-FS expression after MI, which was characterized by up-regulated expression of ACT A, down-regulated expression of FS, and increased ratio of ACT A to FS. In the MI-ramipril group, the expression of both ACT A and type II activin receptors (ActRIIA and ActRIIB) were down-regulated, the expression of FS was increased, and collagen deposition was attenuated. These findings indicate a novel mechanism that may explain the cardiac anti-fibrotic effect of ACE inhibitors and cardiac protection following left ventricular remodeling.

Ventricular remodeling, including cardiomyocyte remodeling, collagen deposition and fibrosis, plays an important role in the pathogensis of HF^15^,^16^. Increased collagen content in the extracellular matrix (ECM) is one of the most important characteristics of myocardial fibrosis^17^,^18^,^19^. Cardiac collagen remodeling occurs not only in the infracted area, but also remote from the infarct site in the post-MI rat heart^20^. Notably, remodeling within the non-infarcted area is the primary contributor to ventricular remodeling^21^. Prior studies have shown that many cytokines are involved in the development of myocardial fibrosis^22^. The role of ACT A in myocardial fibrosis, especially in extracellular matrix production, is similar with TGF-β1. As most members of the TGF-β family, activins mediate biological effects through transmembrane receptor serine/threonine kinases. Activin receptors are divided into ActRI and ActRII^23^,^24^,^25^,^26^, and the latter is subdivided into ActRIIA and ActRIIB^24^,^27^. Activin initially binds to ActRII and then recuits and phosphorylates ActRI. Phosphorylated ActRI activates target genes by activating the Smad pathway^28^,^29^,^30^. FS, an endogenous activin antagonist, can block the activity of activin by preventing activin from binding to ActR^31^,^32^.

Previous studies have reported that ACT A is a potent activator of renal interstitial fiboblasts, could promote cell proliferation, enhance the expression of col I mRNA, and induce the production of α-smooth muscle actin in a rat kidney fibroblast cell line (NRK-49F cells)^33^. ACT A has also been shown to have a mitogenic effect on MC3T3-El cells in an undifferentiated state and modulates the function of osteoblastic cells regulated by FS during differentiation^34^. Several studies have reported ACT A mRNA is up-regulated within 6 hours following carotid injury in rats, and the expression of ACT A has been shown to be greater in arteriosclerotic lesions as compared with non-diseased vessels^35^,^36^. In recent studies, the expression of ACT A has been shown to be increased following myocardial ischemia-reperfusion injury^37^. These results suggest ACT A plays a potent role in cell proliferation, fibrosis, tissue repair, and could enhance the synthesis of extracellular matrix. Our results showed that the expression of ACT A is associated with the synthesis of collagen, suggesting up-regulated expression of ACT A may be one of the predisposing factors for myocardial fibrosis and ultimately heart failure after MI.

FS is an antagonist for ACT A, and is highly expressed in the ovary, pituitary, kideny, heart, and liver. FS binds to ACT A with high affinity, and FS inhibits the biological activities of ACT A by preventing ACT A from binding to its receptors. Previous studies reported that in the soleus muscle, FS protein and mRNA expressions were lower in HF rats after MI^38^. Consistent with these results, we found that myocardial expression of FS in a rat model of HF was lower, indicating the down-regulated expression of FS after MI could enhance the role of ACT A in heart failure and fibrosis. We hypothesize that the antagonistic effect of FS on ACT A could be improved by up-regulating FS expression or exogenous FS and by reversing the imbalance between ACT A and FS, and thus the left ventricular remodeling, heart failure, and myocardial fibrosis could be attenuated. Ramipril, an ACE inhibitor, up-regulated FS expression, which inhibited ACT A from binding to ActRII and ultimately reduced left ventricular remodeling.

In addition to ACT A, Ang II is also an important regulator of cytokine expression involved in myocardial fibrosis. An *in vitro* study has reported that blockade of Ang II type 1 receptor was associated with inhibition of col I synthesis and regression of myocardial fibrosis^39^.

While a limited number of studies have explored the role of Ang II in myocardial fibrosis, the role of Ang II has not been elucidated thoroughly, especially the relationship between Ang II and ACT A. Although recent studies have shown that angiotensin II upregulated activin A in peripheral blood mononuclear cells (PBMNC) *via* activation of NFkB in heart failure^40^, inhibition of NFkB does not completely suppress Ang II-mediated upregulation of activin A in bone marrow-derived mononuclear cells -derived conditioned medium (CM). While ramipril’s effect on the expression of activin A may be related to Ang II, these prior results demonstrate that there could be other contributing mechanisms. The current study demonstrated that increased Ang II in the noninfarcted area of the left ventricle after MI was inhibited by Ramipril. In this study, we found the levels of ACT A in the circulation and myocardial tissue were positively correlated with Ang II expression during ACT A -FS imbalance after MI. We also found the levels of ACT A in serum were positively correlated with the levels of BNP and Ang II, which are two factors related to ventricular remodeling. These results demonstrate that the level of ACT A may have potential as a serological indicator of heart failure.

In summary, Ang II and the ACT A-FS system are involved in heart failure after MI^41^. The interaction of ACT A, TGF-β and could stimulate the secretion of ACT A, creating a positive feedback loop that promotes fibrosis^42^. FS could inhibit the role of ACT A by binding to ACT A and block the interaction of ACT A, TGF-β and the positive feedback loop. The current study demonstrated that an imbalance in ACT A-FS expression occurred after MI, and is characterized by up-regulation of ACT A, which is an important predisposing factor for myocaridal fibrosis and ultimately heart failure.

Futhermore, we provide evidence that the antifibrotic effect of Ramipril is mediated via expression of ACT A-FS. Future therapies may be designed to target this novel mechanism to attenuate left ventricular remodeling during HF after myocardial infarction.

## Materials and Methods

The study was approved by the Ethical Board Review of China-Japan Union Hospital, Jilin University.

### Rat model of heart failure

Female Wistar rats weighing 200–220 g (8 weeks old) were provided by the Experimental Animal Center, Jilin University. The rats were subjected to ligation of the anterior descending branch of the left coronary artery or sham operation during halothane anesthesia (1% halothane in a mixture of one third O_2_ and two thirds N_2_O) as previously described^8^. The rats were fed a standard diet and tap water and kept in groups of 8 rats per cage at room temperature (21 ± 1 °C) under 12-hour light/dark cycle. The animal experiments, procedures, and housing were in accordance with institutional guidelines for the protection of animals used for experimental purpose. Post-operated rats were divided into four groups (n = 6 each group): SO, sham-operated rats receiving 0.5% sodium carboxymethy cellulose (CMC); SO-ramipril group, sham-operated rats treated with ramipril (3 mg/kg/day); MI group, MI rats receiving 0.5% CMC; MI-ramipril group, MI rats treated with ramipril (3 mg/kg/day). All treatment regimens were initiated 7 weeks after surgery and continued for 5 weeks. Ramipril (C7002) was provided by Sanofi-Aventis Pharma. Beijing Co., Ltd.

### Survival Rate

A total of thirty-two Wistar rats were included in the study. As needed throughout this study, halothane was administered for anesthesia (1% halothane in a mixture of one third O_2_ and two thirds N_2_O). Prior to the operation, excessive anesthesia led to the death of two rats. Following the operation, the surviving rats were divided into four groups, SO group (n = 6), SO-ramipril group (n = 6), MI group (n = 9), and MI-ramipril group (n = 9). No rats died in the SO group or SO-ramipril group. Within 24 hours post-surgery, two rats in the MI group, and one rat in the MI-ramipril group died. Within 1 week after surgery, one rat died in the MI group, and two rats died in the MI-ramipril group. Six weeks after surgery, the survival rate in the MI and MI-ramipril groups was 66.7%. Seven weeks after surgery, the drug was administered continuously for 5 weeks, and all 24 rats survived the course of drug administration.

### Hemodynamic measurements

The rats were anesthetized by intraperitoneal injection of 3% pentobarbital sodium (30 mg/kg). A catheter was inserted into the right carotid artery and then further advanced into the left ventricular chamber to record left ventricular systolic pressure (LVSP), left ventricular end-diastolic pressure (LVEDP), and rate of contraction and relaxation (±dp/dt). The systolic blood pressure (SBP), diastolic blood pressure (DBP), and heart rate (HR) were also recorded.

### Tissue Sampling

The rats were euthanized by decapitation, and the hearts were immediately isolated. The heart weight and left ventricular mass were measured, and the ratio of heart weight to body weight (HW/BW) and the ratio of left ventricular mass to body weight (LVM/BW) were calculated.

The infarcted and non-infarcted left ventricular tissue was separated. The non-infarcted left ventricular tissue was cut into two parts along the longitudinal axis of the left ventricle. One portion of the non-infarcted left ventricle was fixed in 4% paraformaldehyde, embedded in paraffin, and sliced into 5-μm sections.

### Morphological changes in the myocardium

Sections (5 μm) were stained by hematoxylin and eosin (H&E) to analyze morphological changes in the myocardium, and Masson’s trichrome to evaluate the degree of myocardial fibrosis according to manufacturer instructions. The expression of ACT A and FS proteins were detected by immunohistochemical staining. The second portion of the non-infarcted left ventricle was stored at −70 °C for determination of protein expression of ACT A, FS and angiotensin II (Ang II), and mRNA expression of ACT A, type IIA and IIB activin receptor (ActRIIA and ActRIIB), FS, and type I and III collagen (col-I and col-III).

### Semi-quantitative reverse transcription polymerase chain reaction

The levels of ACT A, ActRIIA, ActRIIB, FS, col-I, and col-III mRNA in the noninfarcted area of the left ventricle were detected by semi-quantitative reverse transcription polymerase chain reaction (RT-PCR). Total RNA was isolated with TRIzol reagent (Invitrogen, CA, USA), according to the manufacturer’s instructions. cDNA was synthesized from 2 μg of total RNA. PCR for β-actin, ACT A, ActRIIA, ActRIIB, FS, col-I and col-III was performed with the following primers: β-actin (359 bp, NM03114): sense 5′-GCTCGTCGTCGACAACGGCTC-3′, antisense 5′-CAAACATGATCTGGGTCATCTTCTC-3′; ACT A (β_A_ subunit) (726 bp, NM_017128): sense 5′-GGATGTGCGGATTGCTTGTGA-3′, antisense 5′-GACCTTGCCATCACACTCCAA-3′; ActRIIA (361 bp, NM_031571): sense 5′-GACAGAACCAATCAGACTGGTG-3′, antisense 5′-TGTGTGACTTCCATCACCGGAA-3′; ActRIIB (704 bp, NM_031554): sense 5′-GCTGCTGGCTAGATGACTTCA-3′, antisense 5′-GATGTCGGTACATCCAGAAGG-3′; FS (348 bp, NM_012561): sense 5′-GCTGCTGCTACTCTGCCAAT-3′, antisense 5′-GGACCCTTCCAGGTGATGTT-3′; col-I (361 bp, NM_053304.1): sense 5′-AGGGTCATCGTGGCTTCTC-3′, antisense 5′-ACCTTCGCTTCCATACTCG-3′; and col-III (704 bp, NM_032085.1): sense 5′-CTCAAGAGCGGAGAATACTGG-3′, antisense 5′-TGCCACCATCATAGACTAGATTC-3′. The reactions were conducted for 32 cycles with the following cycle conditions: pre-denaturation at 95 °C for 90 seconds, denaturation at 94 °C for 30 seconds, annealing at 58 °C (activin A, at 56 °C) for 30 seconds, and extension at 72 °C for 50 seconds. The final extension was at 72 °C for 10 minutes. The products were separated on 1.5% agarose gel. The band intensity was evaluated by image analysis (ImageMaster VDS, Pharmacia Biotech, USA).

### ELISA

Serum levels of ACT A and the factors affecting left ventricular remodeling, Ang II and BNP, were evaluated using ELISA (ADL, USA) according to the manufacturer’s protocol. Expression of ACT A, FS, and Ang II in the non-infarcted left ventricular myocardial tissue homogenates was also measured. The protein levels in non-infarcted left ventricular myocardial tissue homogenates were measured using a Protein Quantification kit (Dojindo Molecular Technologies Inc, Japan) following the manufacturer’s instruction.

### Immunohistochemcial staining

Immunohistochemical staining for ACT A and FS in non-infarcted left ventricle were performed on the myocardial tissue which was fixed with 4% paraformaldehyde, de-parafinized, rehydrated in a graded series of alcohol solutions, and washed twice in distilled water. The sections were incubated with endogenous peroxidase blocked in 50 μl of 3% H_*2*_O_*2*_at room temperature for 10 minutes. The sections were washed 3 times in pH 7.4, 0.02 mol/L phosphate-buffered saline (PBS) for 3 minutes, and incubated with 2% BSA-PBS at room temperature for 30 minutes. Each section was washed once in PBS, and incubated with either a rabbit anti-rat antibody against ACT A (1:300 dilution; R&D Systems, UK) or a rabbit anti-rat antibody against FS (1:200 dilution; R&D Systems, UK) overnight at 4 °C. The ACT A and FS proteins were assayed with an Ultrasensitive SP kit (Shanghai, China). Sections were counterstained with hematoxylin. Incubation of tissue sections with the IgG from normal rabbit served as a negative control.

### Statistical analysis

All results are presented as mean ± standard deviation (SD). Statistical significance was determined by a bivariate analysis of variance (ANOVA) followed by Dunnett post hoc test. A value of P < 0.05 was considered statistically significant. All experiments conformed to the Chinese Academy of Medical Sciences ethics code of practice.

## Additional Information

**How to cite this article**: Wei, Q. *et al.* Ramipril attenuates left ventricular remodeling by regulating the expression of activin A-follistatin in a rat model of heart failure. *Sci. Rep.* **6**, 33677; doi: 10.1038/srep33677 (2016).

## Figures and Tables

**Table 1:** Morphological and hemodynamic characteristics of sham-operated rats (SO), ramipril-treated SO rats (SO-ramipril), rats with myocardial infarction (MI) and ramipril-treated MI rats (*n* = 6).

Characteristics	SO	MI	SO-ramipril	MI-ramipril
BW (g)	270.63 ± 25.09	285.50 ± 12.50	271.0 ± 20.25	284.13 ± 23.81
HW/BW (mg/g)	3.60 ± 0.19	4.91 ± 0.20***	3.20 ± 0.21	3.96 ± 0.17^†††^
LVW/BW (mg/g)	2.34 ± 0.10	3.84 ± 0.15***	2.29 ± 0.10	2.99 ± 0.11^††^
HR (/min)	418 ± 45.7	406 ± 39.1	415 ± 36.7	428 ± 33.4
SBP (mmHg)	245.1 ± 22.3	132.5 ± 24.6**	189.0 ± 20.8	164.9 ± 20.4
DBP (mmHg)	210 ± 22.1	102 ± 13.4**	141 ± 26.1	145 ± 7.9
LVSP (mmHg)	256 ± 21.5	187 ± 5.6*	243 ± 18.6	201 ± 19.5^†^
LVEDP (mmHg)	46 ± 1.2	69 ± 5.7**	40 ± 4.1	34 ± 5.2^†^
+dp/dtmax (mmHg)	5627 ± 556	3879 ± 435**	5356 ± 421	4573 ± 368^††^
−dp/dtmax (mmHg)	5561 ± 341.2	3531 ± 668.4**	6170 ± 612.7	3346 ± 221.2^†^

BW, body weight; HW, heart weight; LVHW: left ventricular heart weight; HR, heart rate, SBP, systolic blood pressure; DBP, diastolic blood pressure; +dP/dt, maximal rate of rise of blood pressure in ventricle chamber; −dP/dt, maximal rate of rise of blood pressure in ventricle chamber; LVESP: left ventricular end systolic pressure; LVEDP, left ventricular end diastolic pressure. *P < 0.05, **P < 0.01, ***P < 0.001, compared with SO group; ^†^*P* < 0.05, ^††^*P* < 0.01, ^†††^*P* < 0.001, compared with MI group.

**Table 2:** Levels of activin A, Ang II, follistatin, and BNP in the serum and in the noninfarcted area of the left ventricle myocardial tissue homogenate of sham-operated rats untreated (SO) and treated with ramipril (SO-ramipril) and rats with myocardial infarction untreated (MI) and treated with ramipril (MI-ramipril) after myocardial infarction (*n* = 6).

Group	Serum			Non-infarcted area of the left ventricle myocardial tissue homogenate		
	Activin A (pg/ml)	Ang II (mg/ml)	BNP (pg/ml)	Activin A (pg/mg protein)	Ang II (ng/mg protein)	FS (ng/mg protein)
SO	289.98 ± 23.06	2.23 ± 0.14	96.68 ± 4.56	41.06 ± 6.19	231.14 ± 11.23	4.99 ± 0.90
MI	689.51 ± 21.01***	6.14 ± 0.11***	678.12 ± 23.89***	67.81 ± 6.76***	489.12 ± 10.78***	0.31 ± 0.06*
SO-ramipril	296.10 ± 15.77	1.93 ± 0.10	89.01 ± 7.88	45.76 ± 6.88	210.23 ± 10.97	5.36 ± 0.19
MI-ramipril	554.12 ± 14.25^†††^	3.78 ± 0.15^†††^	356.89 ± 17.89^†††^	51.09 ± 4.51^††^	378.21 ± 12.19^††^	6.89 ± 0.96^†^

*P < 0.05, ***P < 0.001 vs SO group; ^†^*P* < 0.05, ^††^*P* < 0.01, ^†††^*P* < 0.001 vs MI group.
